# Promoting Self-Efficacy of Nursing Students in Academic Integrity Through a Digital Serious Game: A Pre/Post-Test Study

**DOI:** 10.3390/nursrep15020045

**Published:** 2025-01-27

**Authors:** Laura Creighton, Christine Brown Wilson, Tara Anderson, Conor Hamilton, Guy Curtis, Christine Slade, Gary Mitchell

**Affiliations:** 1 School of Nursing and Midwifery, Queen’s University Belfast, Belfast BT9 7BL, UKtanderson@qub.ac.uk (T.A.); gary.mitchell@qub.ac.uk (G.M.); 2 School of Psychological Science, The University of Western Australia, Perth, WA 6009, Australia; 3 Institute for Teaching and Learning Innovation, The University of Queensland, Brisbane, QLD 4072, Australia; c.slade@uq.edu.au

**Keywords:** academic integrity, professionalism, artificial intelligence, serious game, nursing student, nursing, self-efficacy, higher education institutions, education

## Abstract

Background: Academic integrity is an important component of nursing education, bridging academic ethics with professional practice. This study evaluated the effectiveness of a co-designed Academic Integrity digital serious game in improving nursing students’ self-efficacy related to academic integrity, academic offenses, professionalism, and artificial intelligence use. Methods: A pre-test/post-test design was employed, using a bespoke questionnaire to assess 303 first-year nursing students’ self-efficacy before and after playing the game. The questionnaire covered five subscales: academic integrity standards, academic offenses, professional values, feedback processes, and AI use in academic work. Results: Statistically significant improvements were observed across all subscales following the intervention, indicating enhanced self-efficacy in understanding and applying academic integrity principles, recognizing academic offenses, demonstrating professional behaviors, utilizing feedback, and appropriately using AI in academic contexts. Conclusions: The Academic Integrity digital serious game has the potential to be an effective tool for enhancing nursing students’ self-efficacy in the areas of academic and professional ethics. This approach shows promise for integrating academic integrity-based education in nursing curricula and preparing students for the ethical challenges of modern healthcare practice. This study was not registered.

## 1. Introduction

Academic integrity forms a central foundation in nursing education, bridging academic skills in higher education with professional ethics in clinical practice [1]. Students who develop a strong understanding and application of academic integrity during their studies are more likely to carry these principles into their professional careers [1]. Globally, nurses consistently rank as the most trusted profession in terms of honesty and ethical standards according to public opinion surveys [2]. This public perception closely aligns with the core values of academic integrity established by the International Center for Academic Integrity, which include honesty, trust, fairness, respect, responsibility, and courage [3]. These core values guide ethical decision-making and behavior in both academic and clinical settings [3].

The incidence of confirmed academic offenses leading to suspected academic misconduct investigations has increased over the past decade, with nursing students, despite their presumed baseline honesty and integrity upon entering their programs, not being immune to these breaches of conduct [4]. This rise in academic misconduct, whereby a student is suspected to have breached the expected academic integrity values, within nursing degrees has raised concerns about its potential impact on professional practice [4]. While plagiarism remains a common academic offense among nursing students in university settings [5], deviations in clinical practice have also been observed, including mis-recording of vital signs, breaching patient confidentiality, and falsely documenting medication administration [4,6]. These findings suggest a potential link between academic and professional integrity among nursing students, raising significant ethical concerns when patient care could be compromised as a result [1,7].

Academic offenses have evolved beyond the traditional scope of cheating and plagiarism to include a broader range of activities that can influence grades across all learning environments, with the definition now including fabrication, collusion, and contract cheating [8]. The COVID-19 pandemic in 2020 introduced additional challenges to education, with the shift to online learning, open-book assessments, and the rising use of Artificial Intelligence (AI) platforms like ChatGPT further complicating the landscape of academic integrity [9,10].

AI has become increasingly integrated into higher education, utilized by both educators and students, with the potential to reshape learning delivery and experiences positively [11,12]. Recent research within higher education suggests that students view ChatGPT as a valuable learning aid, particularly for academic writing, language instruction, and in-class learning [13]. However, this reliance on AI tools raises concerns about academic integrity. Notably, there is a risk that students may become overly dependent on these technologies for all academic work, potentially diminishing critical thinking skills such as problem-solving and original thought, competencies that are highly valued and actively encouraged in higher education [14,15].

The adoption of AI tools in academic settings can inadvertently lead to academic misconduct. This occurs when students overly rely on these technologies, such as using them to generate entire assignments or substantial portions of work without proper attribution or acknowledgment [16]. Such practices not only violate academic integrity principles but also undermine the learning process and the development of critical academic skills [16]. Research indicates that students are often aware of the potential for academic misconduct when using ChatGPT, expressing concerns about the spread of misinformation and issues of fairness [17]. However, many students also believe that the provision of clearer guidelines and targeted education could promote more responsible use of these AI tools in academic settings [18].

Higher academic institutions have adopted a more proactive stance on academic integrity, emphasizing education and knowledge to encourage the avoidance of academic offenses, while resorting to punitive measures only when violations are confirmed. The methods of delivering such education vary, ranging from mandatory online modules [19] to the use of gamification or serious games with educational content [20]. Digital serious games are particularly appealing to contemporary student cohorts, who have grown up in an era of digitalization and widespread device usage. Evidence suggests that serious games can effectively enhance educational content delivery by improving knowledge and empathy in dementia care among pre-registration nursing and pharmacy students [21]. Additionally, they have been shown to promote self-efficacy, increase knowledge, and lead to behavior change, as evidenced by digital serious games aimed at boosting vaccination uptake for influenza and COVID-19 [22]. While the dissemination of academic integrity knowledge alone may not sufficiently reduce the prevalence of offenses, empowering students with both self-efficacy and the knowledge to alter their practices may support the proactive educational approach.

Building on previous research that demonstrated improved motivation for learning about academic integrity through a serious game using validated instruments and focus groups [23], this study takes a further step in evaluating the effectiveness of a co-designed Academic Integrity digital serious game. While the previous study focused on motivation and the viability of serious games as a learning tool, the current research specifically examines the game’s impact on nursing students’ self-efficacy related to academic integrity, academic offenses, professionalism, and artificial intelligence.

Aim: The aim of the study is to engage students in developing both academic skills and knowledge in relation to academic integrity that interlink with their professional values whilst undertaking a nursing degree.

Research Objective:

To determine the impact of an Academic Integrity serious game on the self-efficacy of nursing students on the topics of (1) Academic Integrity, (2) Academic Offenses, (3) Professional Values, (4) Feedback, and (5) Artificial Intelligence.

## 2. Materials and Methods

### 2.1. Ethics

This study was approved by the Faculty of Medicine, Health, and Life Sciences Research Ethics Committee (MHLS 23_120). Participants were not required to provide written or verbal consent for participation in the study. However, they were explicitly informed that their participation in any of the questionnaires was entirely voluntary. Consent was implied when participants accessed the surveys and chose to complete them. Data collection start date 9 October 2023.

### 2.2. Methodology

A quasi-experimental, quantitative pre-test/post-test design was used to compare student confidence and ability in academic integrity, academic offenses, professionalism, and artificial intelligence before and after playing the Academic Integrity serious game. This design was chosen as all students received the link to the game whether they gave consent to participate in the study or not, with the aim to provide equality of learning in the module it was embedded in. The methodological background to this intervention study involved manipulation of the independent variable; therefore, the introduction of the Academic Integrity game was the intervention, and the self-efficacy of the nursing students was the dependent. No randomization was used in a sample population that used a pre-existing group; a cohort of pre-registration year-one nursing students. Pre/post testing was used to assess the changes in self-efficacy over time, in this research project, before and after use of the Academic Integrity game.

A bespoke questionnaire (Supplementary File S1) was designed by the research team to evaluate self-efficacy. The questionnaire was designed to mirror the scenarios in the Academic Integrity game. The sub-sections are academic integrity, academic offenses, professionalism, and use of artificial intelligence, answered by self-report, using a Likert scale with five options: strongly agree, agree, neutral, disagree, and strongly disagree. The questionnaire was developed with reference to international standards on academic integrity and designed by the research team who have expertise in this field, alongside educational experience with nursing students [3,4,23]. The questionnaire was reviewed and revised by a member of the research team with expertise in questionnaire design from the field of psychology alongside an extensive research background in contract cheating [GC]. The questionnaire underwent face validity testing with a sample of nursing students (n = 30) who have previously used the Academic Integrity game as part of their routine teaching in the Professionalism in Nursing (NFM1120) module. The sample of n = 30 nursing students were given the questionnaire and asked to comment using a subjective assessment of the following factors: relevance, formatting, readability, clarity, and the appropriateness for the intended audience. The feedback was recorded using an online form that was anonymous; only minor changes to wording were recommended to add clarity. The Academic Integrity Impact questionnaires were delivered immediately prior to and following participation in the game. The study was conducted using non-probability convenience sampling of all year-one pre-registration nursing students at the School of Nursing and Midwifery, Queen’s University Belfast (n = 340). First year nursing students were chosen as the target population as they are new to academic norms in a higher education setting and at the point of intervention delivery had not yet submitted any assignments. The Transparent Reporting of Evaluations with Nonrandomized Designs (TREND) reporting guideline informed the reporting of this intervention evaluation and is available from the corresponding author on reasonable request.

### 2.3. The Intervention

The serious game was developed using a seven-step co-design process [24] involving nursing students and academic staff across three workshops, as previously reported [23]. The digital game was built by Focus Games Ltd, Glasgow, Scotland and hosted online at https://www.academicintegritygame.co.uk/, accessible as an HTML5 web application compatible with multiple devices. The game’s scenarios cover four key areas: Academic Offenses (simulating a university investigation of academic misconduct), Professional Conduct (demonstrating consequences of skill omissions in a clinical setting), Feedback Utilization (strategies for incorporating tutor feedback), and Study Skills (practical academic preparation, appropriate use of AI and self-care techniques). The Academic Integrity game is an asynchronous resource, with students completing the 45 min gameplay within a specified timeframe. Upon completion, participants receive a certificate verifying their engagement with the educational content.

### 2.4. Consent and Recruitment

All first-year pre-registration nursing students from the September 2023 cohort were emailed by their Director of Education to inform them about this study (n = 316). This was a different sample than the previous evaluation [23]. The email contained an information sheet (Supplementary File S2) and contact details of the research team. The email directed students to their institutional module page in which the questionnaires and game were embedded. It was a requirement that participants tick a box to confirm that they had read the participant information sheet in order to provide consent for taking part in the study. Students were informed that whether or not they chose to participate in the study would not affect the grade associated with the module. Those students who did not consent to participate in the study still were allocated the serious game to play asynchronously in order to have equity in student learning. Withdrawal from the study at any stage was made clear to the participants, information on how to undertake this process was available in the participant information sheet provided prior to the study by the gatekeeper. Embedded within the module platform were the pre/post questionnaires, completed before and after use of the serious game. A student number was provided by the participants in order to pair the pre/post questionnaire responses for analysis.

### 2.5. Data Collection

The pre- and post-questionnaires were completed using Microsoft Forms, these can be viewed in the datasets. The pre-questionnaire recorded demographic details (gender, ethnic group, and education level, and employment status outside of the program). This was followed by a bespoke Academic Integrity Impact questionnaire with five academic integrity-based subscales, each consisting of six items. The first subscale (α = 0.89) measured student awareness of academic integrity standards (e.g., I understand what is meant by academic integrity). The second subscale (α = 0.82) measured student awareness of academic offenses (e.g., I understand how to avoid plagiarism). The next subscale (α = 0.89) measured student awareness of professional values (e.g., I am confident with demonstrating professional nursing behaviors in the clinical setting). The following subscale (α = 0.78) measured student awareness of feedback processes (e.g., I understand how to use a marking rubric to develop my academic study). The final subscale (α = 0.90) measured student understanding of using artificial intelligence within their academic work (e.g., I am confident in my ability to appropriately attribute AI-generated content in my academic work).

### 2.6. Data Analysis

The pre-/post-test questionnaire datasets were matched using the student numbers as an identifier for each study participant. Analyses were conducted in SPSS version 29. The only individuals with access to the data were the research team and the data collected were stored in compliance with the General Data Protection Regulations (GDPR). The data were stored electronically on a password-protected file in an anonymous format, only identifiable by a unique allocated participant number. Storage of the data file on a university computer was in accordance with GDPR (2018).

Likert scale responses for both the pre- and post-test questionnaires were calculated as total scores for each of the sub-scales within. Demographic details of the participant sample were presented using descriptive statistics. Multiple paired *t*-tests were conducted to examine the pre-/post-test changes for each sub-scale of the questionnaire. A Bonferroni correction was applied to the alpha value when determining the significance of the results of the analyses to reduce the risk of false positives associated with multiple comparisons [25], as five comparison analyses were conducted in the study. Alpha (0.05) was divided by this total number of comparisons (5) to give an alpha value of α = 0.01. Results were therefore only considered to be statistically significant if their associated p-value was 0.01 or below.

## 3. Results

In total, 303 participants (Table 1) were recruited to this study in October 2023. Most participants were female (89.4%), white (94.1%), had secondary school level education (84.2%), and were employed part-time (less than 20 h) outside of their university degree program (62.4%). A full list of participant demographics can be viewed in Table 1.

Primary analysis was possible for N = 276 cases out of a total of N = 303 due to missing data. If a participant was missing either pre-test or post-test data, they were excluded from paired *t*-test analyses, but their non-missing data were included in descriptive statistics. Missing data occurred due to a participant not completing either the pre- or post-test measures, or due to participants not supplying a correct identifier (student number) to allow their pre- and post-test data to be matched. Table 2 shows the level of missing data at pre-test, post-test, and analysis.

Paired samples t-tests were used to determine whether there was a statistically significant mean difference between pre- and post-test scores on each of the five subscales (academic integrity, academic offenses, professional values, feedback, artificial intelligence). For all subscales, these differences were statistically significant at the *p* < 0.001 level, i.e., below the Bonferroni-corrected alpha cut-off of *p* = 0.01. Table 3 shows the means, standard deviations, 95% confidence intervals (CIs), and effect sizes (Cohen’s d) for these tests. These results indicate that the intervention had a substantial influence on students’ understanding and self-efficacy across all measured domains.

### Assumption of Analysis of Findings

The five subscales of the bespoke Academic Integrity Impact questionnaire of Academic Integrity, Academic Offenses, Professional Values, Feedback, and Artificial Intelligence all showed statistically significant results following data analysis of the paired results.

The largest effect size was Artificial Intelligence at 1.44, followed by Feedback at 1.23. The smallest effect size was Academic Offenses at 0.70; however, this is still considered to be a medium to large effect. Interpretation of the results would suggest that the intervention of the Academic Integrity game has a positive impact on student self-efficacy across the five sub-scales measured. The most substantial improvements were in the understanding of Artificial Intelligence and ability to give/receive Feedback. While Academic Offenses and Professional Values showed the smallest improvements, the changes are still meaningful. These data suggest that the intervention was effective in improving students’ understanding and skills across various academic and professional domains, with particularly strong effects on AI knowledge and feedback skills.

## 4. Discussion

The integration of digital serious games in nursing education offers a promising approach to enhancing knowledge about academic integrity and improving self-efficacy among students. A prior study by the authors [23], utilizing a serious game intervention focused on academic integrity, demonstrated statistically significant improvements in motivated strategies for learning. Using a 34-item validated Motivated Strategies for Learning Questionnaire [26], the study found that students showed higher internal goal orientation, critical thinking, self-regulation, help-seeking behaviors, and peer learning after playing the game. These findings are consistent with the current study, which demonstrated statistically significant improvements in self-efficacy specifically related to the subdomains of academic integrity, understanding of academic offenses, professional values, feedback, and artificial intelligence in one cohort of 1st year nursing students who received the Academic Serious Game intervention. The high response rate in the prior study, with a sample of 233 students, reflects a similar strong engagement observed in the current study, demonstrating strong interest from participants. Results of the study suggest that Artificial Intelligence and Feedback showed the highest impact. A potential rationale could be that the nursing students are in their first semester of their nursing program and so have never been exposed to information on these topics. The lowest effect, although still showing statistical significance, was academic offenses. This could be explained by students receiving module handbooks for each of the five curriculum modules, as well as a university-specific module handbook in their first week of starting the program. They would be familiar with the process of what happens should you be suspected of or commit an academic or practice offense for that reason; however, the positive outcome following use of the Academic Integrity game may be due to the storyboard-based nature of the subject, whereby a student is taken through the process. To determine if these proposed assumptions are correct, a further aspect of the study would need to be considered as was carried out in the previous iteration of this project by using focus groups and collecting qualitative data.

Several serious games have been developed specifically to address academic integrity. CiteSaga, for example, is a tabletop game designed to enhance citation skills and understanding of academic integrity principles [27]. Evaluated using the “Play with Purpose” heuristic framework, it was found to significantly increase participants’ knowledge of citation styles and improve their overall learning experience [27]. Another serious game, Plagiaruedo, engages players in solving a fictional plagiarism case, requiring them to navigate university departments and submit their findings to a fictional plagiarism detection system [28]. Additionally, the Integrity Games platform offers a series of gamified cases that encourage undergraduate students to reflect on various dilemmas related to academic integrity [29]. This platform emphasizes the importance of understanding the gray areas between outright cheating and acceptable practices, encouraging deeper reflection on integrity issues commonly faced by students [29]. Collectively, these studies highlight the importance of collaborative design and working with others in the development of educational resources. This aligns with the experiences of pre-registration nursing students, who actively participated in the co-design of the present academic integrity serious game [30]. Furthermore, the outcomes reported from existing serious games focused on academic integrity align with the present study, which also demonstrates that improvements in self-efficacy can result from engaging with serious games.

While there has been a growing interest in the use of serious games in higher education to promote academic integrity, there remains a limited number of digital serious games specifically focused on academic integrity [23]. Moreover, few of these games target the nursing profession or are designed for asynchronous learning environments [23]. Asynchronous education has gained prominence in nursing education, particularly in response to the challenges posed by the COVID-19 pandemic [31,32]. This mode of learning allows students to engage with course materials at their own pace, making it particularly suitable for nursing students who often juggle irregular work schedules [33]. A recent scoping review of the international research indicated that asynchronous education could enhance self-efficacy among nurses by promoting self-directed learning and providing flexible access to educational resources [34]. These findings support the current study, which also found positive improvements in academic integrity using asynchronous resources with nursing students. Specifically, the study demonstrates that academic integrity can be effectively enhanced through this approach, which aligns with the growing evidence supporting the benefits of asynchronous learning in nursing education.

This emphasis on asynchronous and gamified learning complements the broader educational perspective on academic integrity. The effectiveness of active learning strategies in promoting academic integrity has long been highlighted, consistently emphasizing the need for approaches that encourage critical reflection on ethical dilemmas rather than relying exclusively on punitive measures within higher education [35]. Similarly, the role of psychological factors, including motivation and self-regulation, in influencing students’ ethical decision-making has also been identified as significant [36]. This established evidence base on academic integrity in higher education supports the implementation of interactive, evidence-based educational interventions designed to enhance understanding and adherence to academic integrity principles, thereby reducing instances of plagiarism, contract cheating, and academic misconduct.

### 4.1. Implications for Nursing Practice

The findings of this study have important implications for nursing practice and education. The demonstrated effectiveness of the Academic Integrity digital serious game in improving nursing students’ self-efficacy related to academic integrity, academic offenses, professionalism, and artificial intelligence use suggests that the serious game is a potentially valuable tool for enhancing ethical conduct in both academic and clinical settings. The improved understanding of academic integrity standards and awareness of academic offenses can lead to more ethical academic practices among nursing students. This enhanced knowledge is likely to translate into more responsible and honest behavior in clinical settings, reinforcing the nursing profession’s reputation for trustworthiness and ethical conduct. In addition, the increased confidence in demonstrating professional nursing behaviors in clinical settings reported by this study indicates that the game could effectively bridge the gap between academic integrity and professional practice. This improved professionalism could in turn lead to better patient care, enhanced teamwork, and more effective communication in healthcare settings. The game’s positive impact on students’ understanding of feedback processes and use of marking rubrics could also encourage a greater culture of continuous improvement and self-reflection among future nurses. This skill is important for ongoing professional development and adaptation to evolving healthcare practices. Furthermore, the improved understanding of appropriate AI use in academic work addresses a critical contemporary issue in education. As AI tools become increasingly prevalent, nurses equipped with the knowledge to use these technologies ethically and effectively will be better prepared to navigate the digital landscape in both academic and healthcare environments. Lastly, the promising results of this digital serious game approach may provide a model for developing similar educational tools across other areas of nursing education. This method of delivering critical content is likely to align well with the learning preferences of contemporary students and could be adapted to address various aspects of nursing practice, from clinical skills to ethical decision-making.

### 4.2. Strengths and Limitations

There is a notable paucity of digital serious games addressing academic integrity specifically for nursing students, who must understand this subject as part of their professional conduct. This novelty highlights the significance of the current study. Several strengths enhance the validity and applicability of its findings. The large sample size (n=303) improves the generalizability of results to broader nursing student populations. A pre-test/post-test design allowed for direct measurement of self-efficacy improvements, supported by a bespoke questionnaire validated through expert input and face validity testing. The study’s focus on contemporary issues, such as the integration of AI into academia, and alignment with international academic integrity standards strengthens its applicability across contexts. However, limitations include reliance on self-reported measures, which may not directly translate to behavior changes, and a single-institution design, which do limit generalizability. Future research should include objective measures, replication across institutions, and longitudinal follow-up to assess sustained impacts on academic and professional conduct. Addressing potential self-selection bias through mandatory participation could also enhance the robustness of findings.

## 5. Conclusions

The Academic Integrity digital serious game demonstrates significant potential as an educational tool for enhancing nursing students’ self-efficacy in academic integrity, professionalism, and ethical AI use. The game’s effectiveness in improving understanding across multiple domains suggests its value in preparing future nurses for the ethical challenges of both academic and clinical environments. While further research is needed to assess long-term impacts and generalizability, this study provides a strong foundation for integrating innovative, digital approaches to ethics education in nursing curricula.

## Figures and Tables

**Table 1:** Participant demographics.

		N	%
Gender	Female	271	89.4%
	Male	22	7.3%
	Non-Binary	2	0.7%
	Missing	8	2.6%
Ethnic Group	White	285	94.1%
	Black	4	1.3%
	Asian	5	1.7%
	Missing	9	3.0%
Highest Level of Education	Secondary School Education	255	84.2%
	University Level Degree	36	11.9%
	University Level Masters	3	1.0%
	Missing	9	3.0%
Employment outside university degree program	Part time (less than 20 h)	189	62.4%
	Part time (more than 20 h)	38	12.5%
	Full time (37.5 h)	6	2.0%
	No extra employment	55	18.2%
	Other	7	2.3%
	Missing	8	2.6%

**Table 2:** Missing data.

	Missing n (%)
Pre-test data	8 (2.60%)
Post-test data	19 (6.27%)
Paired comparisons	27 (8.91%)

**Table 3:** Paired *t*-tests’ descriptive statistics.

Scale	Pre-Mean (SD)	Post-Mean (SD)	95% CIs	Cohen’s d
Academic Integrity	21.63 (4.65)	26.69 (3.12)	4.49 to 5.64 *	1.06
Academic Offenses	24.67 (3.41)	26.97 (2.89)	1.91 to 2.69 *	0.70
Professional Values	24.70 (3.50)	27.06 (2.90)	1.96 to 2.75 *	0.71
Feedback	21.93 (3.57)	26.61 (2.94)	4.23 to 5.13 *	1.23
Artificial Intelligence	18.66 (5.00)	26.30 (3.07)	7.00 to 8.27 *	1.44

* *p* < 0.001.

## Data Availability

The datasets generated and/or analyzed during the current study are available from the corresponding author upon reasonable request.

## Supplementary Materials

The following supporting information can be downloaded at: https://www.mdpi.com/article/10.3390/nursrep15020045/s1, File S1: Academic Integrity Impact Questionnaire; File S2: Participant information sheet.

## Public Involvement Statement

There was no public involvement in any aspect of this research.

## Guidelines and Standards Statement

This study is not a clinical trial and so has no registration of trial number available. This manuscript was drafted against the TREND statement for reporting of intervention evaluation studies using non-randomized designs (https://www.equator-network.org/reporting-guidelines/improving-the-reporting-quality-of-nonrandomized-evaluations-of-behavioral-and-public-health-interventions-the-trend-statement/) (accessed on 17 November 2024).

## References

[1] Guerrero-Dib J.G., Portales L., Heredia-Escorza Y. (2020). Impact of academic integrity on workplace ethical behaviour. Int. J. Educ. Integr..

[2] Girvin J., Jackson D., Hutchinson M. (2016). Contemporary public perceptions of nursing: A systematic review and narrative synthesis of the international research evidence. J. Nurs. Adm. Manag..

[3] International Centre for Academic Integrity (ICAI) (2021). The Fundamental Values of Academic Integrity.

[4] Allen C., Stanley S., Cascoe K., Stennett R. (2017). Academic dishonesty among undergraduate nursing students. Int. Arch. Nurs. Health Care.

[5] Birks M., Smithson J., Antney J., Zhao L., Burkot C. (2018). Exploring the paradox: A cross-sectional study of academic dishonesty among Australian nursing students. Nurse Educ. Today.

[6] Lovrić R., Žvanut B. (2022). Profiling nursing students’ dishonest behaviour: Classroom versus clinical settings. Nurs. Ethics.

[7] McClung E.L., Schneider J.K. (2018). Dishonest behavior in the classroom and clinical setting: Perceptions and engagement. J. Nurs. Educ..

[8] McClung E.L., Gaberson K.B. (2021). Academic Dishonesty Among Nursing Students: A Contemporary View. Nurse Educ..

[9] Adzima K. (2020). Examining online cheating in higher education using traditional classroom cheating as a guide. Electron. J. E-Learn..

[10] Lo C.K. (2023). What is the impact of ChatGPT on education?. A rapid review of the literature. Educ. Sci..

[11] Essien A., Chukwukelu G., Essien V. (2020). Opportunities and challenges of adopting artificial intelligence for learning and teaching in higher education. Fostering Communication and Learning with Underutilized Technologies in Higher Education.

[12] Kuka L., Hörmann C., Sabitzer B. (2022). Teaching and learning with AI in higher education: A scoping review. Learning with Technologies and Technologies in Learning.

[13] Hassan A. (2023). The Usage of Artificial Intelligence in Education in Light of the Spread of ChatGPT. Emerging Trends and Innovation in Business and Finance. Contributions to Management Science.

[14] Črček N., Patekar J. (2023). Writing with AI: University Students’ Use of ChatGPT. J. Lang. Educ..

[15] Putra F., Rangka I., Aminah S., Aditama M. (2023). ChatGPT in the higher education environment: Perspectives from the theory of high order thinking skills. J. Public Health.

[16] Acosta-Enriquez B.G., Arbulú Ballesteros M.A., Arbulu Perez Vargas C.G., Orellana Ulloa M.N., Gutiérrez Ulloa C.R., Pizarro Romero J.M., Gutiérrez Jaramillo N.D., Cuenca Orellana H.U., Ayala Anzoátegui D.X., López Roca C. (2024). Knowledge, attitudes, and perceived Ethics regarding the use of ChatGPT among generation Z university students. Int. J. Educ. Integr..

[17] Famaye T., Adisa I., Irgens G. (2023). To Ban or Embrace: Students’ perceptions towards adopting Advanced AI Chatbots in Schools. Advances in Quantitative Ethnography, Proceedings of the ICQE 2023, Melbourne, VIC, Australia, 8–12 October 2023.

[18] Montenegro-Rueda M., Fernández-Cerero J., Fernández-Batanero J.M., López-Meneses E. (2023). Impact of the implementation of ChatGPT in education: A systematic review. Computers.

[19] Curtis G.J., Gouldthorp BThomas E.F., O’Brien G.M., Correia H.M. (2013). Online Academic-integrity Mastery Training May Improve Students’ Awareness of, and Attitudes toward, Plagiarism. Psychol. Learn. Teach..

[20] Kiryakova G., Angelova N., Yordanova L. (2014). Gamification in Education. https://www.researchgate.net/publication/320234774_GAMIFICATION_IN_EDUCATION/citations.

[21] Craig S., Barry H.E., Carter G., Stark P., Mitchell G., Clarke S., Wilson C.B. (2024). Evaluation of a dementia awareness game for health professions students in Northern Ireland: A pre-/post-test study. BMC Med. Educ..

[22] McConnell H., Duncan D., Stark P., Anderson T., McMahon J., Creighton L., Craig S., Carter G., Smart A., Alanazi A. (2024). Enhancing COVID-19 Knowledge among Nursing Students: A Quantitative Study of a Digital Serious Game Intervention. Healthcare.

[23] Creighton L., Mitchell G., Hamilton C., Stark P., Mclaughlin-Borlace N., Craig S., Slade C., Brown Wilson C. (2025). Integrating Academic and Professional Integrity: A Co-Deisigned Serious Game for Nursing Students. Int. J. Educ. Integr..

[24] Santin O., McShane T., Hudson P., Prue G. (2019). Using a six-step co-design model to develop and test a peer-led web-based resource (PLWR) to support informal carers of cancer patients. Psycho-Oncology.

[25] Bland J.M., Altman D.G. (1995). Multiple significance tests: The Bonferroni method. BMJ.

[26] Soemantri D., Mccoll G., Dodds A. (2018). Measuring medical students’ reflection on their learning: Modification and validation of the motivated strategies for learning questionnaire (MSLQ). BMC Med. Educ..

[27] Bunt L., Bunt B., Pelser-Carstens V., Matthew G. (2024). Play with Purpose: A Heuristic Approach to Evaluating Tabletop Serious Games for Academic Integrity Education. Proceedings of the 18th European Conference on Games Based Learning.

[28] Johnson I., Barclay L. Plagiaruedo*: Teaching of academic integrity through a ‘whodunnit’game (* any likeness to other games is intentional!). J. Learn. Dev. High. Educ..

[29] Goddiksen M.P., Allard A., Armond A.C.V., Clavien C., Loor H., Schöpfer C., Varga O., Johansen M.W. (2024). Integrity games: An online teaching tool on academic integrity for undergraduate students. Int. J. Educ. Integr..

[30] McLaughlin-Borlace N., Creighton L., Mitchell G. (2024). Championing student participation in co-designing digital education resources: A student experience. Nurse Educ. Today.

[31] Singh H.K., Joshi A., Malepati R.N., Najeeb S., Balakrishna P., Pannerselvam N.K., Singh Y.K., Ganne P. (2021). A survey of E-learning methods in nursing and medical education during COVID-19 pandemic in India. Nurse Educ. Today.

[32] Jang S.J., Lee H. (2023). Social jetlag and quality of life among nursing students during the COVID-19 pandemic: A cross-sectional study. BMC Nurs..

[33] Varkey T.C., Varkey J.A., Ding J.B., Varkey P.K., Zeitler C., Nguyen A.M., Merhavy Z.I., Thomas C.R. (2023). Asynchronous learning: A general review of best practices for the 21st century. J. Res. Innov. Teach. Learn..

[34] Kimura R., Matsunaga M., Barroga E., Hayashi N. (2023). Asynchronous e-learning with technology-enabled and enhanced training for continuing education of nurses: A scoping review. BMC Med. Educ..

[35] Curtis G.J., Slade C., Bretag T., McNeill M. (2022). Developing and evaluating nationwide expert-delivered academic integrity workshops for the higher education sector in Australia. High. Educ. Res. Dev..

[36] Tight M. (2024). Challenging cheating in higher education: A review of research and practice. Assess. Eval. High. Educ..

