# Supplementary material for: Promoting Self-Efficacy of Nursing Students in Academic Integrity Through a Digital Serious Game: A Pre/Post-Test Study
Source: Nurs Rep. 2025 Jan 27;15(2):45. doi: 10.3390/nursrep15020045 (PMC11858506; doi:10.3390/nursrep15020045)
Supplement: Supplementary file 1 [file nursrep-15-00045-s001.zip › nursrep-3394703-supplementary.pdf]

Participant Information Sheet

Title of Project:

Supporting student understanding about academic integrity and professional values through a serious educational game: A quantitative evaluation.

Pre/Post-Test Questionnaire

Our teaching team have developed a digital game, that will support development of your academic study skills and avoidance of academic offences.

Serious Educational games are a novel approach to delivering knowledge and learning to undergraduate nursing students.
We are interested to learn how this approach affects your knowledge and attitudes about academic integrity.

We would like to invite you to complete a short Academic Integrity Impact questionnaire about academic integrity and professional values, before and then again after you have participated in the academic integrity game, which is part of your NFM1120 Professionalism in Nursing module.

Please take the time to read through this information before you decide whether you wish to take part.

If you would like to know more about this evaluation, or require more information, please contact Laura Creighton whose details are at the end of this information sheet.

What is the purpose of the study?

The purpose of the study is to learn what how a digital game on academic integrity may influence year one nursing students' knowledge and attitudes about academic integrity and professional values.

QUEEN'S UNIVERSITY BELFAST

Who is doing the study?

The lead researchers involved in this study are Ms Laura Creighton, Lecturer, School of Nursing and Midwifery, Queen's University Belfast (QUB), Mr Conor Hamilton, Lecturer School of Nursing and Midwifery, QUB, Dr Gary Mitchell, Reader, School of Nursing and Midwifery, QUB, Professor Christine Brown Wilson, School of Nursing and Midwifery, QUB and Associate Professor Christine Slade, Honorary Senior Lecturer, School of Nursing and Midwifery, QUB.

Why am I being asked to take part?

You are being asked to take part because you are a student of the module NFM1120: Professionalism in Nursing and as such you will get a chance to play a digital academic integrity game, as part of the module content. We would like to know what influence this game has had on your knowledge and attitudes relating to academic integrity.

What does the study involve?

We will invite you to complete an Academic Integrity Impact questionnaire before and after participating in the academic integrity digital game. The link to these questionnaires will be available on the module page of the NFM1120: Professionalism in Nursing Canvas home page.

The academic integrity impact questionnaire has 24 statements with a scale of strongly agree to strongly disagree across 4 headings; academic integrity, academic offences, professional values and artificial intelligence and will take about 10 minutes to complete.

Do I have to take part?

No, taking part is completely voluntary. If you agree to take part, we will ask you to confirm you have read this information sheet and tick a box to consent on each

online questionnaire. You are free to withdraw from the study at any time without giving a reason. For the purpose of statistical analysis your student number will be linked to the data until the analysis has been undertaken when it will be removed. This will be from the point of the pre-questionnaire until 2 weeks after the post-questionnaire that you will be able to withdraw your data. After that time point it will have been anonymised.

What are the possible advantages of taking part in the study?

There are no direct benefits to you from taking part in the study. However, the information obtained will help provide us with an evidence-base for the modification or continuation of the academic integrity game in pre-registration nursing education.

What are the possible disadvantages and risks of taking part?

There are no direct risks to you from taking part in the study.

Will my participation in the study be kept confidential?

All information about your participation in this study will be kept confidential. We will only ask you to provide us with your student number prior to completing each questionnaire. This is important as it enables the research team to match your responses before and after you playing the game. Your student number will then be removed. We will not collect your names. We will ask for information about you such as; gender, age, ethnicity, 1st language, highest education level and employment status outside of the programme. The information will be stored as an encrypted password protected file on a password protected computer from Queen's University Belfast. The demographic data will be for data presentation tables and not linked to study outcomes. Where fewer than 3 participants fall into a particular cell in the descriptive table, data will be collapsed to protect confidentiality of participants.

What happens when the research study stops?

When the study is finished, we will email the cohort who took part in the game, a one page summary to share the findings of this project. This will be done via the gatekeeper Johanna McMillan. The research team may also disseminate the findings via conference proceedings or publication in a healthcare journal.

What if there is a problem?

If you have any concerns about any aspects of the study, you can contact the Chief Investigator: Ms Laura Creighton using the contact details provided at the end of this information sheet. Should you remain unhappy and wish to make a formal complaint, you can contact the Research Governance Team at Queen's University Belfast.

Who has reviewed the study?

This study has been reviewed by the Faculty of Medicine, Health and Life Sciences Research Ethics Committee.

Further information and contact details

If you would like to talk in more details about any aspects of this research, please contact:

Chief Investigator

Ms Laura Creighton | laura.creighton@qub.ac.uk

Lecturer (Education) School of Nursing & Midwifery, Queen's University Belfast

Thank you for taking the time to read this information sheet

Academic Integrity Impact Questionnaire

This questionnaire will ask about your level of agreement with statements related to your knowledge and confidence with academic integrity, academic offences, professional values, feedback and Artificial Intelligence.

Academic Integrity

1. I am confident I have the knowledge to identify potential academic offences.

Strongly disagree	Disagree	Neutral	Agree	Strongly agree

2. I am confident I have the knowledge to discuss academic integrity with others.

Strongly disagree	Disagree	Neutral	Agree	Strongly agree

3. I have the confidence to use my study skills to adhere to academic integrity standards

Strongly disagree	Disagree	Neutral	Agree	Strongly agree

4. I understand what is meant by academic integrity.

Strongly disagree	Disagree	Neutral	Agree	Strongly agree

5. I understand why similarity reporting software is used

Strongly disagree	Disagree	Neutral	Agree	Strongly agree

6. I am confident I can undertake correct referencing citation.

Strongly disagree	Disagree	Neutral	Agree	Strongly agree

Academic Offences

1. I believe I should report concerns about copying by fellow students to university staff.

Strongly disagree	Disagree	Neutral	Agree	Strongly agree

2. I believe that having good knowledge about academic integrity is important to avoid academic offences'

Strongly disagree	Disagree	Neutral	Agree	Strongly agree

3. I have the confidence to use study skills to avoid academic offences.

Strongly disagree	Disagree	Neutral	Agree	Strongly agree

4. I understand the main processes of fitness to practice following academic misconduct.

Strongly disagree	Disagree	Neutral	Agree	Strongly agree

5. I understand how to avoid plagiarism

Strongly disagree	Disagree	Neutral	Agree	Strongly agree

6. I believe a student could be expelled from an academic programme for academic offences

Strongly disagree	Disagree	Neutral	Agree	Strongly agree

<u>Professional Values</u>

1. I understand the link between academic integrity and professional nursing values.

Strongly disagree	Disagree	Neutral	Agree	Strongly agree

2. I am confident with demonstrating professional nursing behaviours in the clinical setting.

**Strongly disagree**	**Disagree**	**Neutral**	**Agree**	**Strongly agree**

3. I have the communication skills to challenge poorly demonstrated values in others.

**Strongly disagree**	**Disagree**	**Neutral**	**Agree**	**Strongly agree**

4. I have the confidence to know when my behaviours may lead to fitness to practice.

**Strongly disagree**	**Disagree**	**Neutral**	**Agree**	**Strongly agree**

5. I know when to be open and honest about my mistakes in clinical practice.

**Strongly disagree**	**Disagree**	**Neutral**	**Agree**	**Strongly agree**

6. I am likely to use my professional values in the academic setting.

Strongly disagree	Disagree	Neutral	Agree	Strongly agree

Feedback

1.I understand how to use a marking rubric to develop my academic study.

Strongly disagree	Disagree	Neutral	Agree	Strongly agree

2. I am confident in approaching my tutor for feedback about an assessment.

Strongly disagree	Disagree	Neutral	Agree	Strongly agree

3. I believe it is important to seek feedback following assessments.

Strongly disagree	Disagree	Neutral	Agree	Strongly agree

4. I know where to seek guidance following feedback on academic writing style.

Strongly disagree	Disagree	Neutral	Agree	Strongly agree

5. I believe it is my responsibility to seek feedback

Strongly disagree	Disagree	Neutral	Agree	Strongly agree

6. I am likely to seek feedback from my fellow students

Strongly disagree	Disagree	Neutral	Agree	Strongly agree

Artificial Intelligence

1. I am confident in my ability to appropriately attribute AI-generated content in my academic work.

Strongly disagree	Disagree	Neutral	Agree	Strongly agree

2. I understand the ethical considerations associated with using AI-powered tools for my academic study.

Strongly disagree	Disagree	Neutral	Agree	Strongly agree

3. I am familiar with the guidelines for integrating AI-generated content into my assignments or study.

Strongly disagree	Disagree	Neutral	Agree	Strongly agree

4. I know how to ensure that AI-generated content does not compromise the originality and authenticity of my academic work.

Strongly disagree	Disagree	Neutral	Agree	Strongly agree

5. I am aware of the potential consequences of misusing AI-powered tools for academic assignments.

Strongly disagree	Disagree	Neutral	Agree	Strongly agree

6. I believe that using AI-powered tools in my academic study enhances my learning experience.

Strongly disagree	Disagree	Neutral	Agree	Strongly agree
